# Protocol for a Randomized Controlled Trial of Proactive Web-Based Versus Telephone-Based Information and Support: Can Electronic Platforms Deliver Effective Care for Lung Cancer Patients?

**DOI:** 10.2196/resprot.6248

**Published:** 2016-10-26

**Authors:** Christine L Paul, Allison W Boyes, Lorna O'Brien, Amanda L Baker, Frans A Henskens, Ian Roos, Tara Clinton-McHarg, Douglas Bellamy, Glenda Colburn, Shiho Rose, Martine E Cox, Elizabeth A Fradgley, Hannah Baird, Daniel Barker

**Affiliations:** ^1^ Priority Research Centre for Health Behaviour School of Medicine and Public Health University of Newcastle Callaghan Australia; ^2^ Hunter Medical Research Institute New Lambton Australia; ^3^ Hunter Cancer Research Alliance Waratah Australia; ^4^ Cancer Council New South Wales Woolloomooloo Australia; ^5^ School of Medicine and Public Health University of Newcastle Callaghan Australia; ^6^ Distributed Computing Research Group School of Electronic Engineering and Computer Science University of Newcastle Callaghan Australia; ^7^ University of Melbourne Parkville Australia; ^8^ Cancer Network Hunter New England Health New Lambton Australia; ^9^ Lung Cancer National Program Lung Foundation Australia Milton Australia; ^10^ Centre for Clinical Epidemiology and Biostatistics School of Medicine and Public Health University of Newcastle Callaghan Australia

**Keywords:** health information, lung cancer, telephone counseling, psychological distress, randomized controlled trial (RCT), telemedicine

## Abstract

**Background:**

Community-based services such as telephone support lines can provide valuable informational, emotional, and practical support for cancer patients via telephone- or Web-based (live chat or email) platforms. However, very little rigorous research has examined the efficacy of such services in improving patient outcomes.

**Objective:**

This study will determine whether: proactive telephone or Web-delivered support produces outcomes superior to printed information; and Web-delivered support produces outcomes comparable to telephone support.

**Methods:**

A consecutive sample of 501 lung cancer outpatients will be recruited from 50 Australian health services to participate in a patient-randomized controlled trial (RCT). Eligible individuals must: be 18 years or older; have received a lung cancer diagnosis (including mesothelioma) within the previous 4 months; have an approximate life expectancy of at least 6 months; and have Internet access. Participants will be randomly allocated to receive: (1) an information booklet, (2) proactive telephone support, or (3) proactive Web support, chat, and/or email. The primary patient outcomes will be measured by the General Health Questionnaire (GHQ-12) and Health Education and Impact Questionnaire (heiQ) at 3 and 6 months post recruitment. The acceptability of proactive recruitment strategies will also be assessed.

**Results:**

It is hypothesized that participants receiving telephone or Web support will report reduced distress (GHQ-12 scores that are 0.3 standard deviations (SD) lower) and greater self-efficacy (heiQ scores that are 0.3 SDs higher) than participants receiving booklets. Individuals receiving Web support will report heiQ scores within 0.29 SDs of individuals receiving telephone support.

**Conclusions:**

If proven effective, electronic approaches such as live-chat and email have the potential to increase the accessibility and continuity of supportive care delivered by community-based services. This evidence may also inform the redesigning of helpline-style services to be effective and responsive to patient needs.

## Introduction

### Background

Cancer is one of the leading causes of disease-related burden in Australia. Lung cancer is the fourth most incident cancer and approximately 10,300 cases were diagnosed in 2014 [1,2]. Lung cancer mortality rates are relatively higher than other cancer types, with only 6% of those diagnosed with small cell lung cancer surviving 5 years post diagnosis [3]. Lung cancer patients and survivors also report a wide array of physical and psychological issues and, when compared with 6 other common cancer types, report significantly higher levels of clinically significant anxiety and depression [4-6]. Despite the clear evidence-based imperative for providing supportive care to lung cancer patients to maximize their quality of life, this group is under researched in terms of supportive care [7].

Generally, cancer patients report dissatisfaction with the amount and type of information provided regarding management of their health, the failure of health care providers to attend to or offer referral for psychosocial needs, and poor coordination of services [8]. These experiences can exacerbate patients’ suffering. With health care resources stretched to capacity, there is an urgent need to assess the potential benefits of alternative modes of service delivery. These modes may include services provided by community-based organizations rather than hospitals or physicians; and telephone-based or Web platforms rather than the relatively costly and less accessible face-to-face options. However, despite the evidence of their need for support, lung cancer patients are underrepresented in the overall profile of community cancer support service users [9]. Proactive strategies for engaging this vulnerable group with alternative community-based services may provide a valuable opportunity to enhance lung cancer care, particularly in providing information and support in managing the debilitating consequences of diagnoses and treatments.

There is substantial evidence that intensive psychological strategies have been associated with improved psychological health and quality of life in cancer patients [10]. A common example of an intensive strategy is cognitive behavior therapy (CBT) in which adaptive coping elements such as emotional support, positive reframing, planning, acceptance, and support seeking are incorporated into care. A Cochrane review of supportive care interventions including psychotherapeutic interventions and nurse-led counseling improved the emotional, psychological, and physical states (ie, dyspnea) of lung cancer patients [11]. In contrast, relatively little rigorous research has examined the effects of less-intensive forms of supportive care provision for cancer patients [12]. These less-intensive forms of counseling may be delivered by community-based telephone helplines, whereby, unlike hospital-based services, individuals do not receive face-to-face counseling from health professionals familiar with their current care; may have a focus on practical or informational support; and may be a singular encounter.

The Cancer Information and Support (CIS) line is a cancer-specific telephone-based service operated by each state-based Cancer Council in Australia via a national telephone number; the same model operates within the United Kingdom and United States [9,13,14]. The service is staffed by experienced health professionals and provides free, confidential support related to informational, emotional, and practical concerns based on a brief, integrative model of care. A recent review found that telephone-based follow-up care conducted by an experienced nurse was acceptable to patients, cost-effective, and at least equivalent to traditional face-to-face follow-up care in meeting patients’ needs [15]. Hence, telephone delivery means that individualized services can be provided to a broad cross-section of cancer patients in a timely fashion while minimizing cost, logistic, and system barriers [16].

Although telephone-delivered supportive care services, including the CIS, have undergone evaluation of patient use, satisfaction, and acceptability, very little rigorous research has been directed toward understanding the real-world effects of low-intensity models, such as the provision of information, emotional support, and practical support in improving cancer patients’ outcomes [14,17-19]. A systematic review identified only 4 randomized controlled trials (RCT) of similar services with conflicting results reported [20]. One RCT provided evidence of efficacy in that phone-based education and social support delivered by research assistants resulted in reduced mood disturbance compared with mailed education [21]. The remaining 3 trials did not find a significant effect on psychosocial outcomes including distress, anxiety, or depression [22-24]. None of these trials were conducted with lung cancer patients.

While telephone-based support is the traditional mode of delivery of these services, some services are seeking to or have recently included parallel forms of Web support [9,25]. These technology-based approaches are perceived to be accessible, safe, flexible, and anonymous by patients [26-28]. Automated electronic platforms for the delivery of intensive psychological therapies (eg, CBT) have also been found to be effective in reducing anxiety and depression [29]. However, a review by Gustafson et al [30] identified mixed effects when information and support for breast cancer patients was delivered via automated, electronic formats. For community-based services, establishing the efficacy of Web support can provide much needed guidance for deciding if this mode of support should be included as part of the suite of services provided.

In the context of the CIS service, the most appropriate first step for testing Web approaches may be via a proactive and personalized, rather than automated, version of the service through email and live chat. Email counseling involves the patient and counselor exchanging questions and responses at the frequency of their choosing over the Internet. Live chat involves typed interactive conversations occurring in real-time over the Internet. Evidence for online peer-support forums and email is promising with a RCT of a multicomponent Web intervention reporting decreased global severity scores in a sample of 325 breast and prostate cancer patients [31]. However, there is no literature regarding Internet-delivered versions of low-intensity community-based support akin to that offered by the CIS service. For example, a Cochrane review examining the effects of email and Web-messaging between patients and health professionals was unable to establish the benefits of such Web-based platforms due to the lack of high-quality studies identified [32]; relevant to this study, none of the 9 reviewed articles included cancer samples.

This multisite, blinded, patient-RCT will be the first to conduct a robust study of the relative merits of telephone- versus Web-based methods for providing low-intensity information and support to people affected by cancer. Newly diagnosed lung cancer patients will be recruited by health professionals and randomly-allocated on a 1:1:1 ratio to 1 of 3 arms: (1) a printed information booklet, (2) proactive telephone-delivered support, or (3) proactive Web-delivered support. The Consolidated Standards of Reporting Trials of Electronic and Mobile HEalth Applications and onLine TeleHealth is used to describe this study [33].

### Aims

The aims of this study are to identify among a group of newly diagnosed lung cancer patients, if: (1) information and support provided either electronically (email and live chat) or by telephone following active recruitment can produce psychosocial outcomes, which are superior to those achieved by minimal ethical care (a printed *‘Understanding Lung Cancer’* information booklet), and (2) information and support provided electronically can produce psychosocial outcomes, which are comparable to those achieved by a telephone approach.

Intervention effectiveness will be measured by changes in 2 primary outcomes: General Health Questionnaire-12 (GHQ-12) and Health Education Impact Questionnaire (heiQ) scores from baseline to 3 and 6 months post recruitment. Higher GHQ-12 scores represent greater distress; once standardized, higher heiQ scores indicate better functioning as a result of improved self-efficacy and health literacy.

### Hypotheses

It is hypothesized that at 6 months follow-up: (1) those in the 2 experimental conditions (proactive telephone-delivered or Web-delivered support) will have GHQ-12 scores that are 0.3 of a standard deviation (SD) lower than those for the control condition and heiQ scores that are 0.3 of a SD higher than those for the control condition, and (2) those in the proactive Web-delivered support condition will have heiQ scores within 0.29 of a SD of those in the proactive telephone-delivered support condition.

## Methods

### Care Coordinator, Nurse, and Clinician Recruitment

As of June 1^st^, 2016, care coordinators, nurses, and clinicians from 50 health services were committed to participating in the study. A variety of recruitment techniques were used. Care coordinators, nurses, and physicians also received study information with a link to a Web-based expression of interest (EOI) form via several national professional organizations such as: the Lung Foundation Australia; the Clinical Oncology Society of Australia; the Medical Oncology Group of Australia; and the Thoracic Society of Australia and New Zealand. Contacts were also identified through publicly available lists of multidisciplinary teams or through personal connections with research team members. Wherever possible, individuals received a personalized email with an embedded link to the EOI form.

The research team contacted individuals who completed a Web-based EOI and arranged a teleconference to gauge capacity and willingness to participate in the study. No specific exclusion criteria related to the size or location of the health service were applied in order to represent the diversity of rural and urban settings in which lung cancer patients may receive care.

### Study Setting

All interventions will be delivered through 1 state-based CIS service, the Cancer Council New South Wales (CCNSW). In usual practice, clients would be automatically connected to the CIS service in their state of residence. A study-specific telephone number and email address will be set up to permit isolation of study participants from other CIS clients. A directory of state-specific services will be created to ensure participants from areas outside of New South Wales can receive the information relevant to their local areas.

CIS consultants are qualified oncology and/or psychosocial health professionals (ie, nurses, social workers, counsellors). Consultants receive extensive training in supportive care principles and therapeutic communication skills. As part of their ongoing training, consultants routinely participate in clinical supervision and professional development workshops; furthermore, a sample of calls are regularly reviewed by a service manager as part of performance evaluations. A total of 6 CIS consultants will participate and are trained to deliver both the telephone- and Web-based interventions.

**Figure 1:**
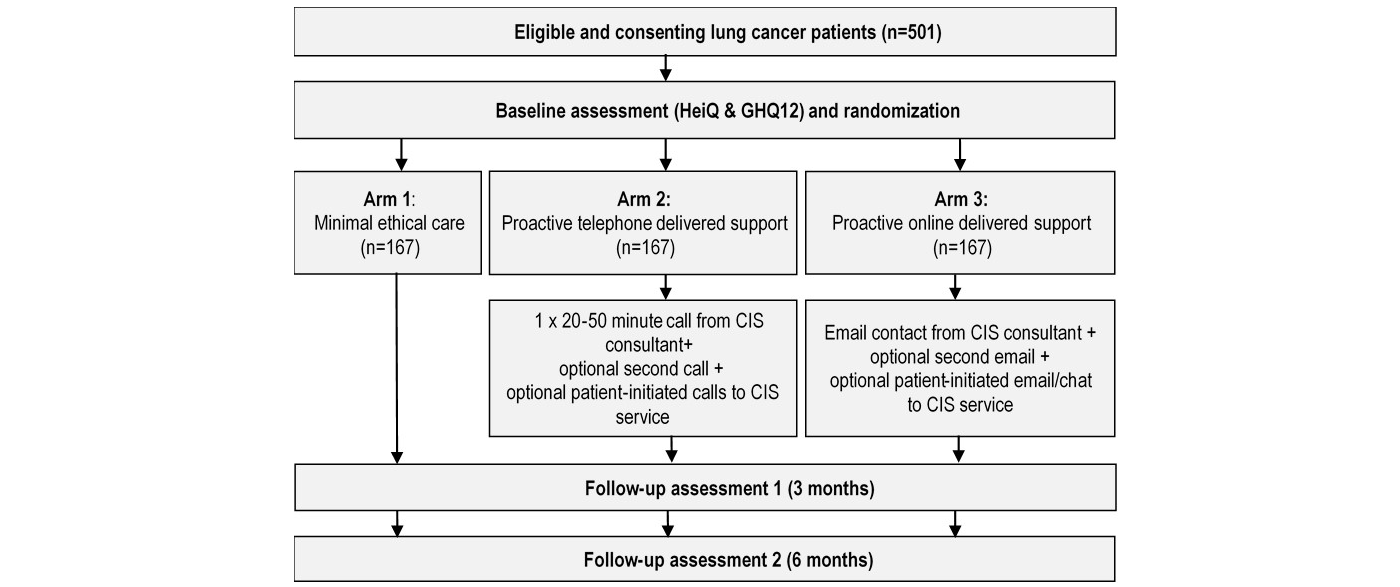
Intervention arms and data collection time-points.

### Participant Eligibility Criteria

Individuals will be eligible to participate if they: are 18 years or older; have a primary diagnosis of any lung cancer type (including mesothelioma); are less than 4 months post diagnosis; have an approximate life expectancy of at least 6 months; and have current Internet access via any type of device including shared access or public access (eg, via a family member or public library).

### Patient Recruitment and Consent Procedure

Eligible lung cancer outpatients will be identified and approached by health professionals (care coordinators, nurses, or clinicians) and will be recruited in 1 of 3 ways:

Full consent process completed in clinic settings immediately: during patients’ appointments, clinic staff or a research assistant (depending on the clinic’s preference) will ask the patient whether they have Internet access, provide study information materials to eligible patients (including a baseline survey), and invite them to participate in the study. Participants will complete a written consent form in clinic and can choose to complete the survey in-clinic or at home.

Consent for further contact completed in clinic settings immediately, with full consent process completed by a research team member: during patients’ appointments, clinic staff will ask whether they have Internet access and if they would be interested in being contacted by the research team regarding the study. Interested patients will complete a consent form that allows the clinic to forward the patient’s contact details to the research team. Once consent forms are forwarded to the research team, patients will be contacted via their preferred mode of contact to discuss possible study participation. Up to 2 follow-up contacts will be made to nonrespondents. Patients who would like to participate will complete a separate consent form, which will be posted along with the baseline survey.

Full consent process completed outside of the clinic setting by a health professional: health professionals, typically care coordinators, will mail information booklets and make follow-up telephone calls to patients as part of standard care procedures. As part of these standard contacts, study materials (information statement, consent form, and baseline survey) will be included in the mail-out package, and the health professional will discuss the study when making calls to patients.

### Randomization Process and Blinding

Stratified block randomization will be completed by the research team using a Web-based random number generator program immediately upon receiving a completed consent form. All participants will complete the baseline survey prior to randomization. Randomization will be by patient, with analyses accounting for clustering of outcomes by CIS consultant. The randomized list of patients will then be given daily to the CIS service to contact participants using the allocated approach method (mailed booklet, telephone call, or email/live chat). Due to the nature of the interventions, patients and CIS consultants will not be blinded to the allocation arm; health professionals will not be informed of participants’ allocation. Those responsible for data analysis and interpretation will be blind to group allocation. There is no foreseeable circumstance in which revealing a participant's group allocation to data analysts would be required.

### Interventions

Participants will be randomized to 1 of 3 arms: (1) a booklet from the Cancer Council which contains the CCNSW CIS service details (minimal ethical care (control)), (2) phone call(s) from the CCNSW CIS service (proactive telephone-delivered support), or (3) email(s) and live chat consultation from the CCNSW CIS service (proactive Web-delivered support). See Figure 1 for brief description with data collection time-points.

#### Arm 1: Minimal Ethical Care (Control)

Patients will be mailed a booklet (“Understanding Lung Cancer”) from the CCNSW. The booklet will contain the CIS service telephone number and email address. Although lung cancer accounts for 9% of all new cancers diagnosed, lung cancer patients account for only 1% of calls received by the CIS service. Therefore, it is not expected that a high proportion of the control group will contact the service independently. Any potential contamination via calls to the CIS service from this group will be identified using questions in the follow-up survey and by reviewing internal CIS records.

#### Arm 2: Proactive Telephone-Delivered Support

The CIS model is tailored to callers’ needs rather than following a manualized protocol as may be the case for delivery of some therapies such as CBT. The patient consent form will request contact details and preferred times to call. The research team will provide this information to the CCNSW CIS service and patients randomized to this intervention arm will receive a 20-50 minute outbound call from a trained consultant within a few days of the CIS service receiving their contact details. The CIS consultant will make multiple attempts (up to 5 calls made at varying times and weekdays) to establish contact with the patient. Following the initial discussion, the CIS consultant will also offer an additional, subsequent call-out. Following the completion of the 2 proactive calls from the CIS, patients in this group can initiate further contact if desired.

##### Call Content

Call content will reflect usual care and will follow the CIS model, in which the call is guided by patients’ individual needs with 3 main types of support available: informational, emotional, and instrumental. Emotional support involves communication of caring and concern, and is argued to reduce distress by improving self-esteem and encouraging the expression of feelings [34,35]. Informational support is thought to enhance perceptions of control by reducing confusion and providing patients with strategies to cope with their difficulties [34,35]; in addition to the verbal information exchanged over the course of a call, specific examples of the informational support provided by the CIS includes: reference to Web-based videos and Web seminars; provisions of paper-based information packages; and access to a cancer service directory. Instrumental support involves the provision of tangible goods such as transportation, money, or physical assistance and leads to a decrease in feelings of loss of control [34,35]. Specific examples of instrumental support offered by the CIS service includes: linking callers with services in their local area; financial grants; transportation grants; and subsidized accommodation. CIS service manuals detailing the 3 main types of support are available upon request. Repeated contact with the same CIS consultant is preferred but not always achieved as a matter of course.

#### Arm 3: Proactive Web-Delivered Support.

Participants allocated to this intervention arm will be contacted within a few days of randomization via email and a hard copy letter. The letter and email will explain the available electronic options for support (email delivered or Web-based typed live chat) and provide the study-specific Web-link needed for intervention access. Participants will be able to use either or both modes of electronic contact according to their preference. If participants have not engaged with either electronic option within 2 weeks of allocation, subsequent telephone calls (up to 5 calls made at varying times and weekdays) will be conducted by the research team to confirm the participant has received the information and to provide additional instructions if needed.

Usual CIS service content (as described above) will be provided via typed rather than spoken communication, using email and Web-based chat. Equivalence of telephone- and Web-delivered content and quality will be examined via 5-10 simulated clients using standardized vignettes. These telephone and Web conversations will be transcribed. The transcriptions will BE reviewed by senior research team members with the content compared with a quality checklist developed in collaboration with CIS representatives according to their current evaluation practices. This process will occur at 3 time-points in the intervention phase and will also serve as a fidelity measure. To ensure the majority of consultants are included in this equivalence test, it may be necessary to complete up to 10 simulations.

CIS consultants will receive training and a detailed manual on the features of the Web system, including ways to convey emotional support, such as empathetic responses using typed text. This manual is available upon request. Multimedia Appendices 1 and 2 provide screenshot examples of the instructions and simulated Web-based chat conversation between a member of the research team and CCNSW consultant. Following their first electronic session, participants will be offered an additional email contact from the CIS service. Similar to the other intervention arm, participants in this group can initiate further electronic contact if desired. Continuity of care (ie, contact from the same consultant) may not always occur. Any potential contamination via calls to the CIS service from the Web-support group will be identified using questions in the follow-up survey and by reviewing internal CIS records.

##### Measures

The baseline and follow-up measures listed below will be collected via pen-and-paper surveys; this data will be securely stored according to approved procedures. The follow-up points for patients will be 3 and 6 months post recruitment, as approximately 60% of lung cancer patients have a life expectancy of less than 12 months [2]. Participants who do not return a survey within 2 weeks will receive a reminder letter and an additional survey package. A research team member will telephone nonresponders 2 weeks following the first reminder letter. All survey variables, including data collection time-points, are listed in Table 1.

**Table 1:** Study data and associated variables collected at each study time-point.

		Time-point		
Study data	Variables	Baseline	3 months	6 months
**Primary outcomes**				
	GHQ-12; heiQ	X	X	X
**Secondary outcomes**				
	SCNS-34^a^ subscales (health systems and information; patient care and support)	X		X
**Process measures**				
	Five items exploring contact and satisfaction with CIS^b^ services; perceived level of consultant skill; and use of specific CIS services (emotional; information; instrumental)			X
**Demographic characteristics**				
	Age at diagnosis; sex; Aboriginal or Torres Strait Islander origin; health insurance coverage; employment status; post code; marital status; highest level of education attained; primary language spoken at home; and concession card holder	X		
**Emotional adjustment**				
	The Mental Adjustment to Cancer Scale [36]	X		
**Disease/treatment**				
	Date of cancer diagnosis; cancer type; other comorbid conditions; surgery and treatments received; and history of mental health treatment prior to cancer diagnosis	X		
	Current extent of cancer; surgery and treatments received; and instances of missed prescriptions in previous week		X	X
**Smoking history**				
	Current smoking status; previous referral and uptake of smoking cessation assistance; and smoking quit date	X		
	Smoking status within the last 6 months			X
**Social support**				
	Medical Outcomes Study-Social Support Survey [37]	X		X
**Illness appraisal**				
	Brief Illness Perception questionnaire [38]	X		X
**Health service utilization**				
	Stanford Health Care Utilization tool [39]			X

^a^SCNS-34: 34-item Supportive Care Needs Survey.

^b^CIS: Cancer Information and Support.

##### Primary Patient Outcomes

The GHQ-12 is a widely used, self-report screening measure of general psychological distress [40]. The 12-item measure takes 2 minutes to complete and assesses an individual’s perception of their health in terms of their ability to: play a useful part; make decisions; overcome difficulties; enjoy normal activities; face problems; and to feel confident, worthwhile, and happy [41]. The time-frame of the GHQ-12 covers the last 4 weeks and items are scored using a 4-point scale (“better than usual,” “same as usual,” “less than usual,” and “much less than usual”) [42]. Half of the items are worded positively, and the other half negatively. Items can be scored using either a Binary scale (0-1, maximum score = 12) or Likert scale (0-3, maximum score = 36), with a higher score indicating higher psychological distress [42]. The GHQ-12 has excellent internal consistency (Cronbach’s alphas above 0.82 for cancer patients) and test-retest reliability [41]. The measure has also been validated in the general Australian community and with cancer populations, including patients with a history of lung cancer [41-44].

Participants who indicate severe levels of distress (scores > 20 on the GHQ-12) at either the baseline, 3, or 6 months survey will be mailed a letter encouraging them to discuss their feelings with their doctor. Contact details of available support services will also be provided.

The heiQ is an Australian-developed tool for assessing the efficacy and impact of health education and self-management programs for people with chronic diseases [45]. Its 42 items are closely aligned to the nature of the CIS service and map to 8 domains: (1) health-directed behavior, (2) positive and active engagement in life, (3) emotional well-being, (4) self-monitoring and insight, (5) constructive attitudes and approaches, (6) skill and technique acquisition, (7) social integration and support, and (8) health service navigation. Respondents indicate the degree to which they agree or disagree with each item on a 4-point scale. Standardized subscale scores (from 1-4) are calculated, with higher scores indicating better functioning.

The heiQ was developed using Structural Equation Modelling and Item Response Theory. It has demonstrated reliability and validity among people with a wide range of chronic diseases and demographic characteristics and sensitivity to change as a result of intervention [45,46].

##### Secondary Patient Outcome

Two subscales of the 34-item Supportive Care Needs Survey (SCNS-SF34) will be used to assess unmet needs [47]. The scale assesses cancer-specific perceived needs across 5, factor analytically derived domains. The 2 relevant domains for this study are: (1) health systems and information, and (2) patient care and support [47]. Respondents indicate their level of need for help over the last month on a 5-point Likert scale. Standardized domain scores ranging from 0 to 100 can be calculated. The SCNS-SF34 has Cronbach’s alphaS greater than 0.86 for each subscale, and is moderately correlated with other measures of psychosocial morbidity [47].

##### Patient Process Measures

Process data will include: number of contacts made to and received from the CIS service; utilization of specific information and support services; acceptability of the information and support provided; and perceived skill level of the CIS consultant. All participants will provide this information at 6 months. Excepting initial telephone calls to participants who do not engage with one of the Web-delivered support options within 2 weeks of group allocation, no additional strategies will be used to increase use of the telephone or Web arms as uptake rates (with consideration of differential use by demographic characteristics) is an important indicator of the intervention acceptability.

##### Antecedent and Moderating Factors

Participants will complete additional survey items to account for sociodemographic, disease and treatment, and social support characteristics, which may moderate the intervention effects. Table 1 outlines these survey items, along with all study data items, and the time-point at which this information will be collected.

### Sample Size

The sample size calculation is based on the post-hoc contrasts. The comparison of each intervention group with minimal ethical care is a superiority analysis, whereas the comparison of the 2 intervention arms of the study is a noninferiority analysis.

The study aims to recruit 501 subjects, and therefore complete data will be available for approximately 375 patients (125 per arm) at 6 months with an estimated 25% (125/501 participants) lost to follow-up. This sample size will provide the study with more than 80% power to detect a difference between each of the intervention groups and the control group of 0.35 SD at an alpha level of 0.01 assuming the correlation between baseline and follow-up scores is at least 0.7. This sample size will also provide the study with 90% power to claim noninferiority in the comparison between the 2 interventions if the true underlying difference between the groups is 0.29 SDs for either the heiQ or GHQ-12. A meta-analysis of Web-based emotional support reported an average effect size of approximately 0.4 across 15 studies [48]; similarly, previous research found modest effect sizes of 0.3-0.5 on the heiQ in self-management interventions in Australian and hospital-based samples [49,50]. The age group and gender of nonparticipants will be compared with that of participants to assess nonconsent bias.

### Statistical Analysis

Primary analysis will compare scores on the GHQ-12 and 5 key heiQ dimensions (emotional support and well-being; monitoring and insight; constructive attitudes; skill acquisition; health service navigation) at a 6-month follow-up across the 3 groups. Differences between treatment groups on each of GHQ-12 and heiQ scores at the 6-month follow-up will be tested using analysis of covariance. The outcomes in the models will be the GHQ-12 score and heiQ score of interest at 6 months, the main predictor of interest will be treatment group and the baseline value of the GHQ-12 score and heiQ score will be included as covariates. Three post-hoc contrasts will be carried out; the first 2 of these will compare separately each of the intervention groups with the control group and the third will compare the proactive telephone-delivered support with the proactive Web-delivered support. To account for Type I error in the post-hoc analyses, Bonferroni corrections will be applied.

## Results

It is hypothesized that participants receiving telephone or Web support will report reduced distress and greater self-efficacy than participants receiving booklets. Furthermore, individuals receiving Web support will report heiQ scores within 0.29 SDs of individuals receiving telephone support. Participant recruitment is underway and will conclude in September 2017.

This study has been approved by the Hunter New England Human Research Ethics Committee (NHMRC Committee Code: EC00403; Reference No. 14/05/21/4.03); the University of Newcastle Human Research Ethics Committee (NHMRC Committee Code: EC00144; Reference No. H-2014-0240); and, the Cancer Council of New South Wales (NHMRC Committee Code: EC00345; Reference No. 291). The study has also been approved by local research governance committees at each participating health service. This trial was registered with the Australian New Zealand Clinical Trials Registry (ACTRN12615000932561) and received funding from the National Health and Medical Research Council as a Partnership Project.

## Discussion

### Trial Outcomes

Lung cancer patients often experience poorer prognosis, more severe physical effects, and more pronounced psychosocial distress than patients with other major cancers. A highly accessible and sustainable source of personalized community-based support would be invaluable for cancer patients and may minimize the need for more intensive and costly hospital-based services. This proposed trial aims to address 3 key issues that are largely unaddressed within the current literature: (1) how to engage patients who may benefit from, but underutilize, the service, (2) whether the model of low-intensity information, support, and referral is effective in improving relevant psychosocial outcomes in the ‘real world’ context, and (3) whether Web-based modes of support are acceptable to, and beneficial for, patients.

The proposed combination of active recruitment to the CCNSW CIS service, and the availability of Web-based options in the present study, has the potential to greatly increase the accessibility and continuity of supportive care for cancer patients. A positive outcome for this trial will be to produce an evidence base for redesigning the CIS service to be both effective and responsive to patient needs, in line with the national health reform’s core principle of patient-centered care and increasing focus on eHealth options [51]. This evidence will also be applicable to a number of international organizations who provide community-based support services based on the CIS model.

### Relevance to Other Community-Based Support Services

The findings from the rigorous examination of the efficacy of low-intensity information and support models of the CIS service are likely to be applicable to, and thus inform, other telephone services that provide similar support to many Australians. These important services, such as Lifeline, Salvo Care Line, Kids Help Line, Mensline, Beyondblue, SANE, Dementia Helpline, Hepatitis Helpline, and Stroke Helpline, are often the foremost services that are widely available to people experiencing a range of distressing and traumatic experiences, and therefore contribute significantly to the mental and social fabric of Australia.

## Appendix

Multimedia Appendix 1 Introduction screen and directions for proactive Web-based delivered support.

Multimedia Appendix 2 An example of a simulated chat conversation providing instrumental and emotional support.

## Abbreviations

CBT: cognitive behavior therapy
CCNSW: Cancer Council New South Wales
CIS: cancer information and support
EOI: expression of interest
GHQ-12: general health questionnaire
heiQ: health education and impact questionnaire
RCT: randomized controlled trials
SCNS-SF34: 34-item supportive care needs survey
SD: standard deviation
