# Supplementary figures and images for: Protocol for a Randomized Controlled Trial of Proactive Web-Based Versus Telephone-Based Information and Support: Can Electronic Platforms Deliver Effective Care for Lung Cancer Patients?
Source: JMIR Res Protoc. 2016 Oct 26;5(4):e202. doi: 10.2196/resprot.6248 (PMC5103105; doi:10.2196/resprot.6248)

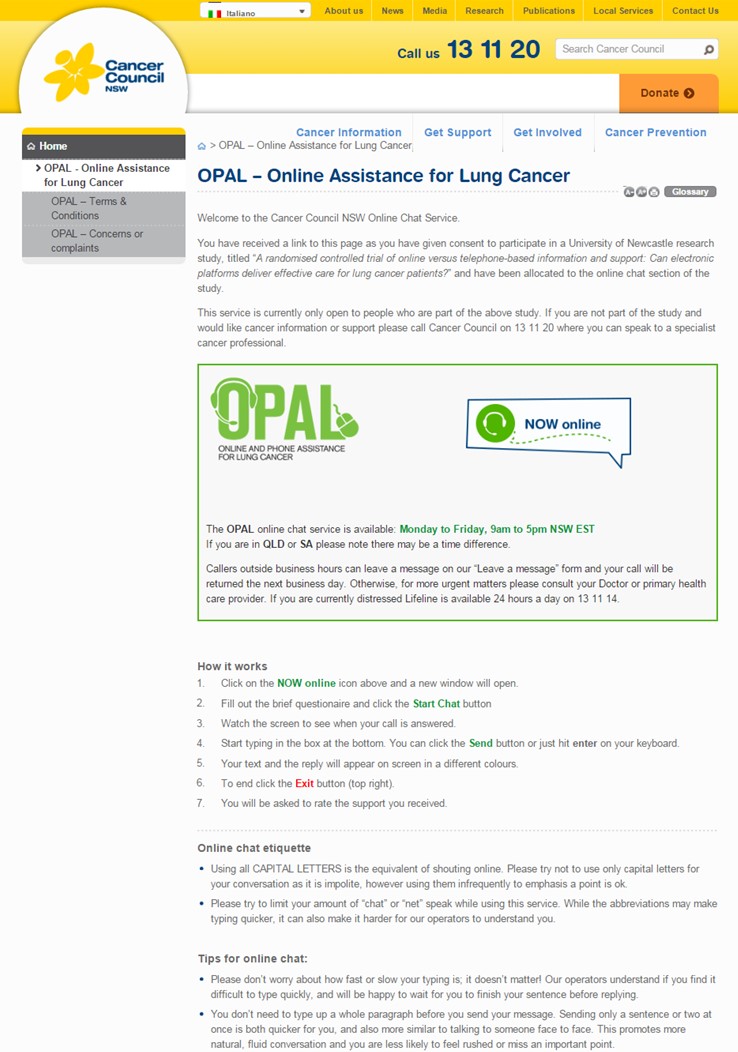

Supplement: Multimedia Appendix 1 [file resprot_v5i4e202_app1.jpg]

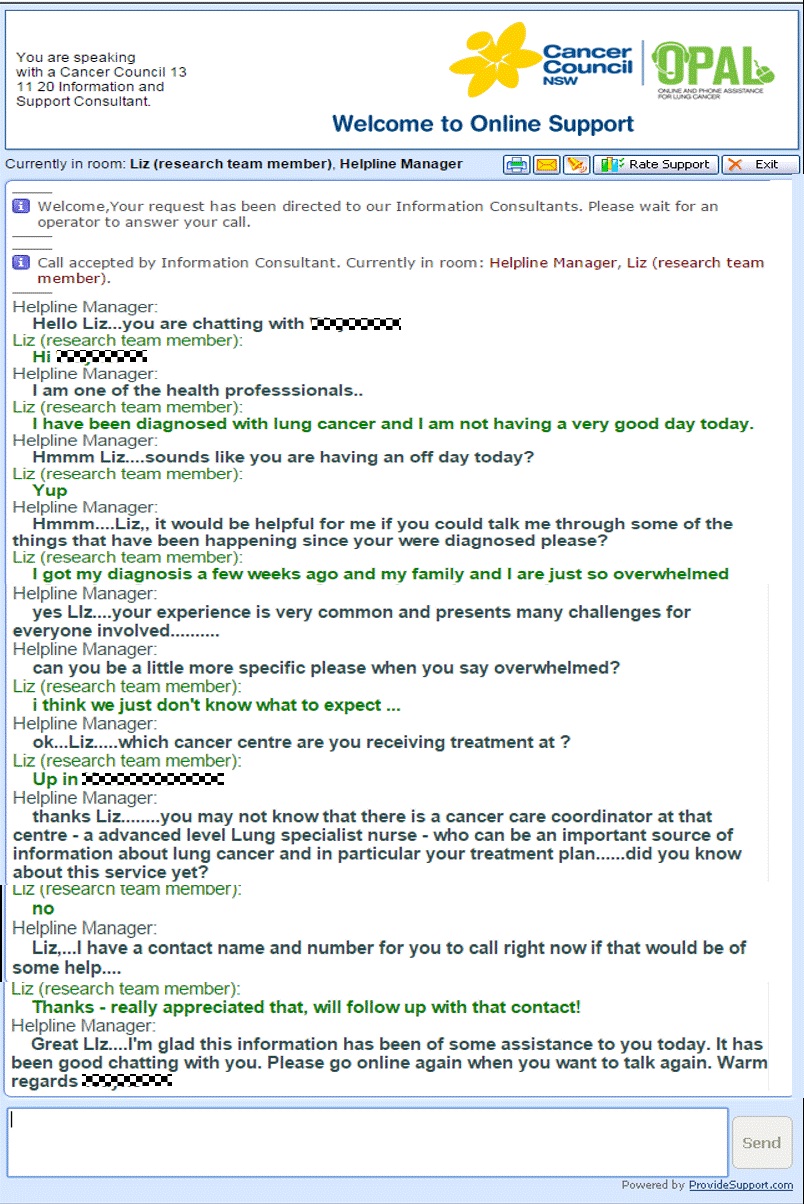

Supplement: Multimedia Appendix 2 [file resprot_v5i4e202_app2.jpg]
